# Patient and public involvement in research: the need for budgeting PPI staff costs in funding applications

**DOI:** 10.1186/s40900-023-00424-7

**Published:** 2023-03-25

**Authors:** Anna De Simoni, Tracy Jackson, Wendy Inglis Humphrey, Jennifer Preston, Heather Mah, Helen E. Wood, Emma Kinley, Laura Gonzalez Rienda, Carol Porteous

**Affiliations:** 1 Wolfson Institute of Population Health, Asthma UK Centre for Applied Research, Queen Mary University of London, London, UK; 2 Usher Institute of Population Health Sciences and Centre for Medical Informatics, Asthma UK Centre for Applied Research, University of Edinburgh, Edinburgh, UK; 3 Institute of Life Course and Medical Sciences, University of Liverpool, Liverpool, UK; 4 Wolfson Institute of Population Health, Queen Mary University of London, London, UK

**Keywords:** Patient and public involvement, Community engagement, Research, Health

## Abstract

**Background:**

Patient and Public Involvement (PPI) groups are becoming more established as collaborators with academic researchers and institutions to ensure that research is important and relevant to end users, and to identify areas that might have ethical considerations, as well as to advise on solutions. The National Institute for Health and Care Research UK Standards for Public Involvement in Research embody best practice for PPI, including support and learning opportunities that build confidence and skills for members of the public to play an invaluable and mutually productive role in research. However, the pivotal role of research and professional services (management and administrative) staff within academic institutions for sustaining and making this involvement successful is often overlooked.

**Main body:**

It takes significant effort to develop and sustain effective PPI in research. The six UK Standards for Public Involvement highlight the need for consistent, inclusive, well-governed and mutually respectful working relationships to sustain effective PPI contributions in health research. Productivity across a team of lay and academic members requires organisation and experience of implementing these standards by a dedicated PPI team, yet advice on PPI finances is usually focused on costs for patient panel members, and budgets in funding applications rarely consider the wider PPI team behind this involvement. As an exemplar, we reflect on how the Asthma UK Centre for Applied Research (AUKCAR) has developed a dedicated PPI Platform, with guidance for how PPI should be embedded throughout the research lifecycle, and detailed information to support the costing of PPI in funding applications. AUKCAR’s work with established researchers, as well as Early Career Researchers and PhD students, is at the heart of a campaign to raise awareness of the importance of PPI in effective research planning.

**Conclusion:**

Focusing attention on the staff behind best practice involvement in health research may stimulate a much-needed discussion to ensure flourishing PPI capacity, with significant patient and public benefit. With adaptation, the PPI expertise within AUKCAR can be translated more widely.

## Abstract (summary)

Patient and Public Involvement (PPI) is important for high-quality research. It makes research more relevant to patients, and makes the results more useful for the health service. To make patient involvement effective, we need skilled staff with experience of involving lay members in research, as well as engaging researchers in PPI activities. There is little guidance about staff time needed to recruit and support lay members and researchers properly. This means that we still do not understand the true cost of including patients and the public in research, and we often under cost this in funding applications. As an example, we reflect on how the Asthma UK Centre for Applied Research (AUKCAR) has organised staff to support for its patient involvement. We give some thoughts on how to cost PPI staff time in funding applications. By focusing attention on the team behind the lay volunteers, we hope to encourage a much-needed discussion about the support involved, and deliver more patient benefits. The AUKCAR experience can be adapted to other research topics and contexts.

## Background

The necessity and importance of Patient and Public Involvement (PPI) in health, public health and social care research has been well established [1–3]. The United Kingdom (UK) National Institute for Health and Care Research (NIHR), and increasingly many other funders, have a requirement for PPI to be included in their funding programmes. NIHR and other UK funding bodies have created guidelines and resources to support and facilitate researchers in including meaningful PPI in their research [4–6]. In 2022, funders, sponsors and regulators, as well as other organisations involved in the delivery of research, have signed the Shared Commitment to Public Involvement [7], stating:

*We will*:

- *Listen to and learn from the people and communities we involve and apply and share that learning*
- *Build and share the evidence of how to involve the public and the impact this has*
- *Support improvements in equality, diversity, and inclusion in public involvement*
- *Promote the UK Standards for Public Involvement* [8].

*We will embed this commitment into the decision-making processes of our organisations*”

None of these activities can be achieved without skilled and supported PPI staff who have expertise and experience in collaborative PPI partnerships. Establishing effective PPI in research is not a quick or straightforward task. At the outset of establishing a PPI group, a significant time commitment is required from lay members, research and professional services staff in research organisations. The focus of this article is on the vital role of these staff for establishing and maintaining effective and collaborative ways of working, and on budgeting their costs in funding applications to ensure their involvement is sustainable.

While ad hoc time-limited PPI groups run by individual researchers for individual studies are common, increasingly researchers are looking for PPI input prior to seeking funding that can then be utilised throughout the research process and as part of the dissemination which may happen after the research has ended. Often, researchers rely on established sustainable PPI networks to support these wider requirements.

## Main text

**Fig. 1:**
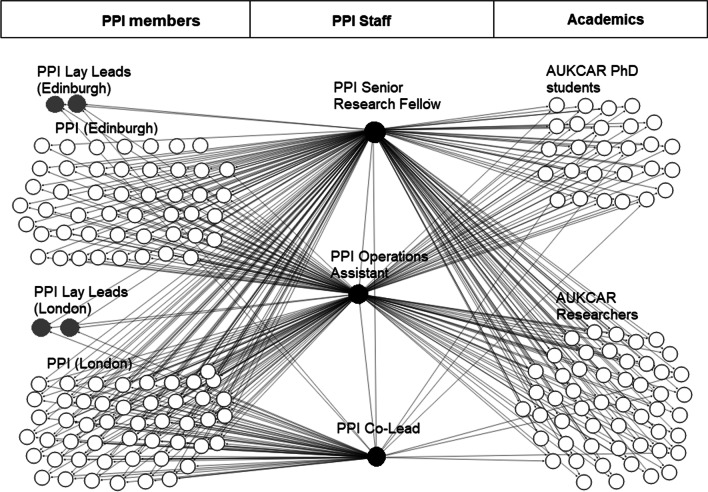
PPI staff interactions with PPI members and academics in AUKCAR. Circles represent individuals. Interactions are represented by lines connecting circles, and can be any of: face-to-face, email, telephone, virtual meetings, social media. Interactions between academics and PPI members are mediated by PPI staff. The workload of PPI staff increases with the number of PPI members and academics, as illustrated by the number of lines between PPI staff and stakeholders

Mathie et al. [9] have recently drawn attention to the ‘invisible’ work of PPI leads and their important role in facilitating PPI activities. The role of PPI staff members (often called PPI leads, managers or officers) is variable across differing institutions and projects, ranging from establishing PPI strategies for large research projects, to administration of the PPI group and its finances. However, PPI staff members often have the job of establishing PPI groups/panels and networks, and developing and sustaining relationships with PPI panel members, which underpin much of the PPI activities. PPI staff members, particularly those that support PPI activities of a research centre, institute or across multiple projects, oversee and monitor requests for involvement in research and dissemination. This ensures that work is planned and evenly distributed amongst a range of lay members, whilst enabling patients and public to have the required support to engage fully with research activities (see Box 1 and Fig. 1). PPI staff are also expected to be experts in planning PPI activities suitable to different study designs, and tailoring support for researchers to increase researcher knowledge and understanding of the intricacies and management of PPI activities.

**Box 1:** Summary of PPI staff tasks

A team of research and professional services experts is required to manage:	
(1) initial recruitment of PPI lay members; providing opportunities to all members to ensure equal chances of involvement;	
(2) Collection and registration of PPI preferences for engagement, including how they want to be paid for their work (e.g., vouchers or more formal contracts);	
(3) Track record of involvement of different PPI lay members in a range of studies to monitor workload and avoid overburdening;	
(4) Provision of relevant training;	
(5) Reimbursement of PPI time, including set up and management of procedures to do so;	
(6) Ongoing contact with PPI lay members, including feedback (e.g., on successful or unsuccessful funding bids; successful or unsuccessful candidates for studentships and early career research roles) to ensure that relationships are maintained for future studies;	
(7) Coordination of lay involvement in research studies once funded;	
(8) Organisation of meetings or regular catch-ups with the research and wider team at all stages in the research cycle; and	
(9) Ongoing recruitment to the lay team to keep up with natural turnover and ensuring PPI members’ diversity

Good interpersonal skills are paramount to bring out PPI voices on equal terms with the scientific team, to enable lay members to speak, for example, in situations when digital barriers may make it harder for PPI members not familiar with these tools. Other important 'soft skills' are collaborative working, communicating effectively, providing appropriate advice and guidance on involvement opportunities, managing group/power dynamics between people and researchers, understanding the organisational and systematic issues of research, translating these into lay terminology, and finally addressing physical/emotional burden from involvement. Such skills are also essential for supporting and encouraging researchers’ interaction with PPIs. These are not taught skills but learned and co-developed through mutual learning with PPI.

Importantly, PPI staff also maintain records of PPI group membership and contributions, regularly monitor preferences in terms of areas of interest and time commitment, ensure compliance with GDPR, as well as consistency of PPI norms and values among researchers. Although guidance on how to ensure compliance with regulations can be shared across PPI networks, thus reducing the average cost, training and continuous professional development underpin PPI staff work, as their involvement is essential for all the points of liaison between researchers seeking PPI input and members of the public seeking involvement in research.

Most lay members go through a process of ‘acquiring a confident voice’, and it is best practice to offer and promote support and learning opportunities to support this. The PPI staff team is a vital part of this process of support and familiarisation. PPI staff enable the creation of a relaxed atmosphere in meetings where new volunteers feels at ease. Progressively and with gentle encouragement from PPI staff, volunteers start to see that their opinion is being heard and is taken on board. PPI staff then communicate the effect/impact of their input on the project, and share research outputs. PPI staff also enable the interaction and sharing of experiences between lay volunteers themselves. Slowly, a relationship of trust is established between PPI staff and volunteers, which is the basis for building future collaborations. Relationship building can also be achieved by regular communication on ongoing activities and PPI opportunities. Staff time and input are then required to maintain the expertise and continued growth of a PPI group by keeping up with members’ turnover.

Alongside these development and support processes, lay members may at any time have a change in health or other personal circumstances that means they are no longer able to offer their time, or may want to move on after a period of contribution. Therefore, PPI staff engage in ongoing recruitment of new people to the group to ensure its continuation, sustainability and ensuring diversity of its members.

Much PPI staff work is devoted to engaging with researchers, including PhD students, throughout the whole research cycle: (a) reminding them of the value of having early input to new research ideas and proposals, and co-creation where possible; and (b) ensuring that the PPI throughout the planned study is appropriate and properly funded. PPI staff organize and run regular meetings to facilitate engagement between PPIs and researchers, from the time they consider making a funding application, up to the dissemination of findings and policy writing once the research has been completed.

For accurate PPI cost analysis, PPI staff time requirements should be appropriately itemised. If a PPI platform is to be established and maintained for a range of research studies at different stages of development, the infrastructure for this in the form of staff support and processes needs to be maintained, necessitating an ongoing funding stream (or careful coordination of multiple funding streams). While the NIHR have a requirement for PPI staff to be included on funding applications, with regard to costs for this, guidance is lacking and limited to suggesting “This role should be a budgeted and resourced research team member” [10] Jinks [11] and colleagues suggest that “sustaining PPI in research is a complex interplay of clarity of purpose, defined roles and relationships, organised support and a robust infrastructure that is well-funded.”

In the subsequent section we describe the infrastructure and funding in the AUKCAR PPI Model.

## Budgeting for PPI staff costs

### The AUKCAR PPI model

The Asthma UK Centre for Applied Research (AUKCAR) is a UK-wide network of world-leading academics and partners, working collaboratively to improve treatment and care for people living with asthma [12]. The establishment of a UK PPI Forum for Asthma Research was incorporated as a central part of the AUKCAR’s vision from its inception in 2014. The PPI staff at AUKCAR are a mix of research and professional services staff. The group started with one academic lead, three co-lay leads and a research management lead. Lay members actively participated in the AUKCAR management committee, core research programmes and cross-cutting workstreams. There was recognition at the outset of the organisational infrastructure required for effective PPI, including training for lay members in research and advocacy, and the need to build in sustainability with resources in each new research study.

In eight years, the Centre has developed a Patient Advisory Group (PAG) of over 100 members of the public—asthma patients, relatives or carers of asthma patients—and the “PPI team” has grown to a PPI Senior Research Fellow, a PPI co-Lead and a PPI Research and Operations Assistant and four co-lay leads, alongside the wider management team (see Fig. 1). The PPI Senior Research Fellow co-produces research with lay members and facilitates PPI research discussions. They are supported by the Research and Operations Assistant to coordinate the PAG meetings, inputs and reimbursements according to NIHR guidelines. Having research backgrounds, the PPI Senior Research Fellow and co-lead are able to greatly assist and support PPI members by explaining and translating research documents into lay language, helping lay members to understand how research is conducted, particularly at grant application stage. A PPI Working Group (consisting of Senior Research Fellow, PPI Operations Assistant, PPI co-lead and PPI lay leads) meets every 2–3 months to discuss issues, challenges and opportunities, and provide guidance to the Centre management team.

A variety of methods are used by PPI staff to engage with existing PAG members and researchers, including telephone, email, face-to-face/remote meetings, and social media. The workload of PPI staff increases with the number of PPI members and researchers seeking PPI input, as illustrated by the number of links between PPI staff and stakeholders in Fig. 1.

Together, this PPI leadership team is responsible for co-developing and spreading a common set of PPI norms and values within the Centre, using best practice as set out in the NIHR Standards for Public Involvement. In addition, the PPI team works closely with colleagues in the AUKCAR Communications/Engagement team with lay leads and PAG contributing to external engagement strategies about research findings. Where research results would contribute to policymaking, PAG and lay leads make suggestions and add their comments to written documents, as equal members of the research team. When there are issues around governance, these are raised at regular project meetings which the lay leads attend, to ensure their equal contribution to resolution.

**Fig. 2:**
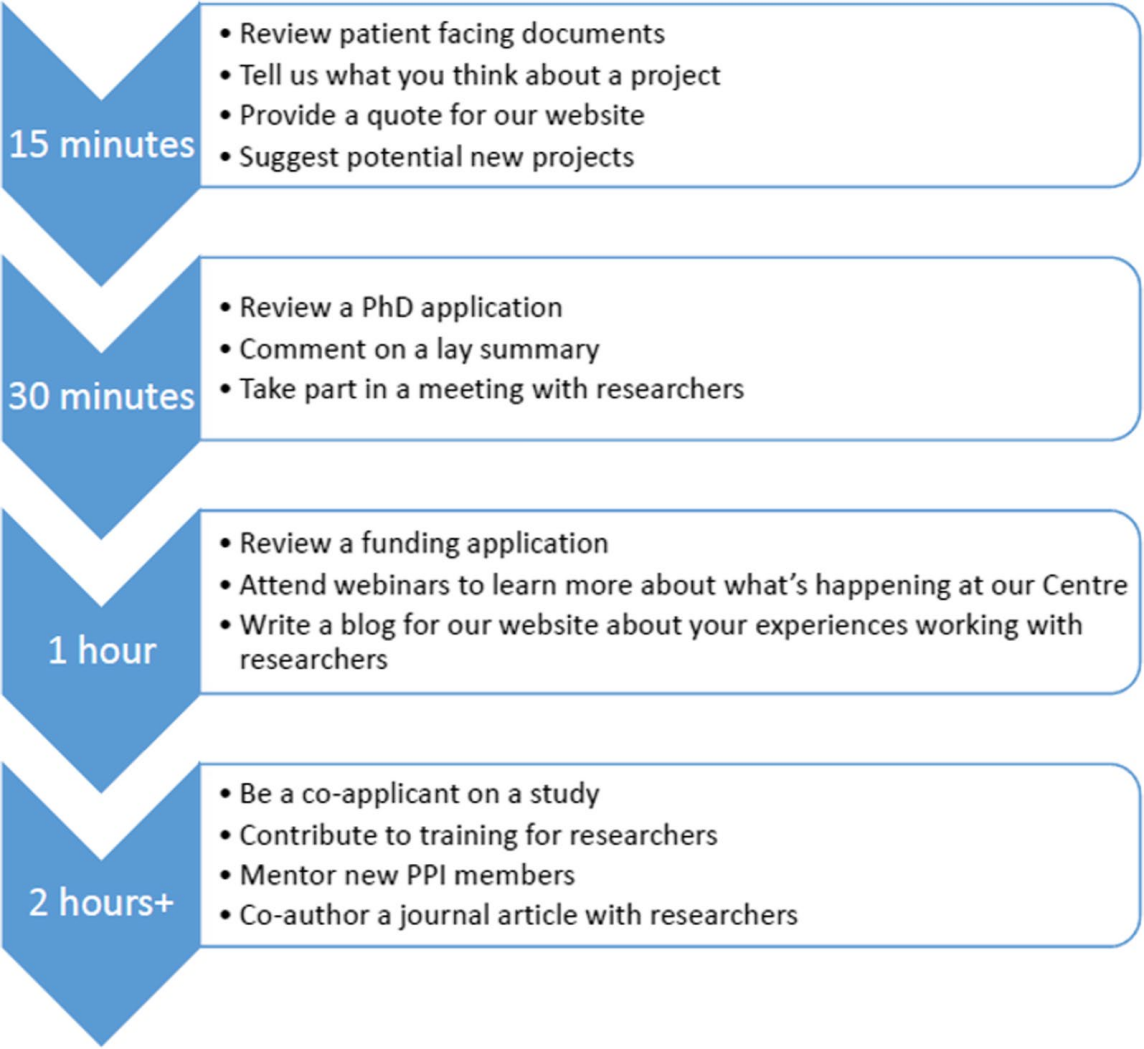
PPI engagement preferences, developed together with AUKCAR PPIs. The times are indicative and may differ based upon PPI experience, personal expertise, or impairments/disabilities

Ongoing infrastructure funding from Asthma + Lung UK over these eight years has proved invaluable in sustaining the development of the AUKCAR PPI model. This has enabled PPI support at both pre-funding and dissemination stages to a range of projects and programme grants, as well as PhD studentships without sufficient budget or expertise to establish their own dedicated PPI group. The culture that has been developed in the Centre is about including PPI throughout the research cycle, from developing research priorities, designing research activities, applying for funding, and undertaking the project, to analyzing the results, dissemination, implementation and evaluation. The AUKCAR PPI staff team also works closely with other respiratory research projects in the UK and internationally to share learning, ideas and best practice to support future development. As an increasingly known and valued PPI network, academic groups external to the Centre are increasingly contacting the AUKCAR PPI team to engage with our pool of volunteers. Provided the staff and lay members have capacity and there is PAG interest in contributing, other research groups can utilize this resource in return for reimbursement, according to UK Standards for Public Involvement^8^ (See Box 2 and Fig. 2 for our suggested PPI involvement times and ways).

**Box 2:** Range of PPI engagement activities according to PPI time preferences

When they join the AUKCAR PAG, lay members are not required to commit to a specific contribution; all levels of involvement are welcome. Examples of involvement opportunities include reviewing funding applications, providing feedback on patient-facing documents, inputting on website design, research steering group membership, co-application on project grants, co-authoring research articles, and presenting at conferences. PAG members will have differing interests, levels of experience/expertise and time available to contribute to such activities. Along with PAG members, we have co-produced a scheme of different PPI engagement activities (Fig. 2) according to members’ time/resources. PPI staff engage with each PAG member to establish their individual preferences, maintain a record of these, and invite their involvement accordingly. This framework is also helpful with recruiting new PAG members, giving concrete examples of PPI tasks that they could get involved with, according to their preferences and availability of time

Since its creation about 9 years ago, the PPI Platform has helped around 90 internal and external researchers, PhD students and other bodies. The involvement of the PPI volunteers in all areas of AUKCAR research has progressed gradually, as the size and expertise of the volunteer cohort and staff grew. To give an example of the volume and diversity of input provided, between January 2021 and January 2023 the AUKCAR PPI Platform has been involved in 27 grant applications (both internal and external, offering support, advice and having an input from early stages through to submission); 18 PhD student requests for assistance with their projects; regular monthly feedback to academics presenting research projects at any stage, from developing ideas to results dissemination at meetings organised in London; co-production of a PPI library of documents (such as a guide for AUKCAR researchers to involve PPI in research and collaborations, PPI costing documents for grant applications, and timeline guidance to ensure PPI members are given adequate time to contribute effectively); input to Optimum Patient Care on the development of the International Severe Asthma Registry (ISAR); input to the shortlisting and interview process of 8 PhD and Early Career Researchers’ posts; contribution to an ‘Impact for Engagement’ course at the University of Edinburgh; co-production of a lecture and a video recording for the MSc in Data Science in Health and Social Care and Q&A session with volunteers; and engagement with the AUKCAR annual scientific meeting. Additionally, there have been PPI-driven research projects, whose results are currently under review in peer scientific journals.

### PPI staff costing suggestion in funding applications

PPI staff time required for organising PPI in research projects is dependent on many variables, such as the type and size of the project and the type of involvement planned. The PPI plan needs to fit the budget available from the funding organisation, but the research planning also needs to consider properly the importance of PPI staff and ensure that there can be genuine and meaningful involvement at all stages of the research process.

In AUKCAR, we have developed some guidance to help researchers budget for PPI staff team costs (Table 1). To calculate PPI staff costs we used project management forecasting techniques, estimating the number of meetings to be held during a study, the duration of each and the preparation required, average number of PPI members for each activity, time to communicate with lay members for each meeting, attendance of the Research Fellow at project meetings and project PPI reporting requirements. For smaller grants (< £250 k) and the hour/daily rate, costs listed in Table 1 account for the hours spent on PPI activities. Variables such as size of the project, who the funder is, what the researchers want to achieve, expected PPI activities throughout the duration of the project, travel, consumables etc., are considered on an individual basis.

**Table 1:** Guidance developed based on AUKCAR PPI work

Project	PPI research fellow/PPI senior research fellow	PPI admin
Programme grants (> £1 m)	0.2–0.5FTE	0.2–0.5 FTE
Large projects (> £250 k)	0.2 FTE	0.2 FTE
Small projects (< £250 k)	Agree a number of days or hours for the project based on the PPI plan	

Published guidance is available on what costs to consider when budgeting for PPI activities. NHS England, in its bite size guide to PPI budgeting, [13] has a comprehensive list of what ‘resources’ should be provided for and does include budgeting for administrative support, but does not specifically mention staff costs, or include details about costings. The NIHR INVOLVE’s budgeting tool [14] has a much more detailed framework and includes a ‘cost calculator’ that can be used to work out the actual costs of involvement for individual studies. This tool mentions implications for research staff time taken in supporting members of the public involved in PPI activities and the need for this time to be costed, as well as including a section on staff costs. There is need of further structuring such costs. The AUKCAR guidance for budgeting PPI staff costs may be a starting base to stimulate discussion for future improvements of PPI budgeting tools. The creation of sustainable infrastructures for PPI begins with an appropriate team: without skilled PPI research and administrative staff to support all aspects of the PPI process, effective lay input cannot happen.

## Conclusions

We acknowledge that PPI costs are often included within ‘core’ costs of infrastructure bids, such as the NIHR Biomedical Research Centres (BRCs) [15] or the NIHR Applied Research Collaborations (ARCs) [16]. For those who are not part of large well-funded streams of established funding infrastructure, there is a need for proper costing of PPI staff to help create sustainable PPI activities within smaller centres or groups. The key roles and skills needed to facilitate PPI activities have been acknowledged elsewhere. This expertise should be valued as a fundamental part of research delivery, and should be appropriately and adequately costed in grant bids. The long-term benefit of properly costed PPI is sustainable, well-supported PPI that has a greater likelihood of achieving impact.

If research teams cannot sustain a stable PPI team, then lay input must begin from scratch with each newly-funded project, rather than creating an ecosystem of mutually-beneficial, trusted relationships. Many funders require PPI to be conducted prior to an application. Without sustainable PPI models, the capacity to engage fully with lay members at the pre-application stage is almost impossible.

AUKCAR PPI was costed as part of the original Centre infrastructure, with a core funding budget covering both PPI members and PPI staff. New grant applications supported by the Centre require the inclusion of appropriate PPI staff costs, to ensure sustainability. As such, the AUKCAR has achieved demonstrable impacts through PPI.

Our guidance is designed to allow for the ongoing development and maintenance of an established, well-trained and experienced PAG, as a resource available to all members of a research centre. PPI staff budgets are indicative and would need tailoring on an individual project basis. Nevertheless, these are essential items to list in PPI budgets in funding applications.

We believe this is a model that could be adopted—with modifications—by other research centres, academic institutions or charities internationally.

## Data Availability

Not applicable.

## Abbreviations

PPI: Patient and public involvement
NIHR: National Institute for Health and Care Research
AUKCAR: Asthma UK Centre for Applied Research
